# Modulating salience network connectivity through olfactory nerve stimulation

**DOI:** 10.1038/s41398-025-03500-6

**Published:** 2025-08-21

**Authors:** Carina Heller, Maria Geisler, Nicolas L. Mayer, Annabelle Thierfelder, Martin Walter, Thomas Hummel, Ilona Croy

**Affiliations:** 1 Department of Psychiatry and Psychotherapy, Jena University Hospital, Jena, Germany; 2 Masonic Institute for the Developing Brain, Institute of Child Development, University of Minnesota, Minneapolis, MN USA; 3 Department of Pediatrics, University of Minnesota, Minneapolis, MN USA; 4 Department of Psychological and Brain Sciences, University of California, Santa Barbara, CA USA; 5 German Center for Mental Health (DZPG), Partner Site Jena-Magdeburg-Halle, Jena-Magdeburg-Halle, Germany; 6 Center for Intervention and Research on adaptive and maladaptive brain Circuits underlying mental health (C-I-R-C), Jena-Magdeburg-Halle, Germany; 7 Lab for Autonomic Neuroscience, Imaging and Cognition (LANIC), Department of Psychosomatic Medicine and Psychotherapy, Jena University Hospital, Jena, Germany; 8 Smell & Taste Clinic, Department of Otorhinolaryngology, University Hospital Carl Gustav Carus, Technische Universität Dresden, Dresden, Germany; 9 Department of Clinical Psychology, Friedrich Schiller University Jena, Jena, Germany; 10 Department of Psychotherapy and Psychosomatic Medicine, University Hospital Carl Gustav Carus, Technische Universität Dresden, Dresden, Germany

**Keywords:** Depression, Molecular neuroscience, Physiology

## Abstract

Depression is associated with reduced functional connectivity within the brain’s salience network and its strengthened interactions with the default mode network (DMN). Modification of this clinical pattern is challenging. Leveraging the direct neural pathways from olfactory processing regions to the salience network, we explored the effects of electrical stimulation of the olfactory mucosa on brain connectivity. In a randomized, blinded within-subject design, 45 healthy individuals received olfactory or trigeminal nerve stimulation followed by resting-state fMRI. Olfactory stimulation resulted in a significant increase in functional connectivity between the salience network and the piriform cortex – a primary olfactory structure. Importantly, this stimulation increased functional connectivity within the salience network and weakened connectivity between the salience network and the DMN. These findings suggest that olfactory stimulation may modulate connectivity patterns implicated in depression, offering a novel potential minimal invasive therapeutic strategy. However, as these results were obtained from a healthy cohort, further studies are required to evaluate the efficacy in individuals with depression.

## Introduction

Anhedonia, defined as the diminished capacity to experience pleasure from rewarding stimuli, is a core symptom of depression. It is characterized by a reduction in interest and motivation to sustain activities that would normally be enjoyable. In severe cases of depression, individuals are unable to maintain occupational or social functioning or even carry out basic daily activities such as getting out of bed. Anhedonia and lack of motivation are associated to reduced activity in the brain’s salience network [1, 2], a group of interconnected brain regions responsible for detecting and responding to salient stimuli amidst irrelevant background information [3]. The network integrates interoceptive and exteroceptive inputs to evaluate emotional significance in the anterior insula [4], while the anterior cingulate cortex (ACC) coordinates appropriate responses across sensory, motor, and association cortices [4–6].

In addition to diminished activity in response to rewarding stimuli, the salience network of depressed individuals exhibits a less orchestrated interplay. Abnormal patterns during resting state, especially within the ACC, have been proposed as a neural classifier for depression [7]. Almost a decade ago, two comprehensive reviews of resting-state functional connectivity in depression highlighted significant alterations within the salience network [8, 9], including decreased connectivity in the bilateral anterior insula which was found to be associated with the severity of depression [10]. This is further supported by a recent large-scale study involving over 600 patients, which demonstrated relative hypoconnectivity within the salience network [11], potentially driven by reduced myelination of salience network tracts in depression [12].

In addition, depression is also marked by hyperconnectivity between the salience network and the default mode network (DMN), both during resting state [10] and when performing tasks related to salience processing, such as responding to smiling faces [13]. This hyperconnectivity was further correlated with severity and decreased glutamate levels in the perigenual ACC [14].

Modifying this clinical pattern using brain stimulation techniques remains challenging. Regional synchronization during resting state was found to increase for theta activity within salience network components such as dorsal ACC while decreasing beta power in anterior DMN structures such as the medial prefrontal cortex (MPFC) [15]. Two studies, involving 91 and 81 patients, respectively, investigated effects of electroconvulsive therapy and found no significant impact on within-salience-network hypoconnectivity or salience-to-DMN hyperconnectivity [8, 9]. However, a study of 23 patients reported an increase in salience-to-DMN connectivity following stimulation [16]. One potential explanation may be the relatively broad effects of an unspecific, brain wide stimulation during electroconvulsive therapy.

An alternative, more regionally confined, approach to targeting salience network function may involve olfaction, given its strong anatomical overlap with the salience network, which likely stems from the co-evolution of these structures [17, 18]. The neural intertwining of olfaction and emotion processing was first reported by McLean, who described the sense of smell as a key part of the “visceral brain” [19], later termed the limbic system [20]. Olfactory processing begins with olfactory receptor neurons located in the upper part of the nasal cavity (olfactory mucosa), which transmit signals to the olfactory bulb, the primary relay station for olfactory information. From the olfactory bulb, signals are sent to the primary olfactory cortex, including the piriform cortex and amygdala [21]. Hence, olfactory stimulation reaches the amygdala within only two neurons. Almost as direct is the connection to the salience network with the anterior insula and the ACC, which both function as secondary olfactory structures and are involved in the hedonic perception of odors [22].

Based on the anatomical connection, McLean already proposed a link between the olfactory system and depressive states [19]. This hypothesis has since been confirmed. Hyposmia – the reduced ability to perceive smells – is associated with impaired emotional perception and processing [23]. Moreover, both acquired and congenital anosmia correlate with increased depressive symptoms [24]. Conversely, major depressive disorder is often characterized by reduced olfactory function across multiple domains, including odor threshold, identification, discrimination, and central processing [24–26], and by reduced volume of the olfactory bulb [24, 25, 27, 28]. Causal support for the olfaction-depression link comes from animal studies, showing that bulbectomized rats (those with surgical removal of the olfactory bulb) exhibit depression-like behaviors, neurotransmitter alterations [29], and amygdala fiber degeneration [30].

The close interplay between emotion, salience and olfaction may bear a potential for improving salience network functioning and emotional responses through olfactory system stimulation. We were inspired by previous research, which demonstrated that electrical stimulation of the olfactory nerve can modify resting-state connectivity. In that study, 18 participants received low-voltage stimulation of the upper nasal tract, followed by a resting-state fMRI scan. The results showed increased connectivity within the primary olfactory structures [31], however the authors did not investigate areas beyond the primary olfactory cortex. Building on this previous work, we sought to determine whether electrical stimulation of the olfactory nerve, compared to stimulation of the intranasal trigeminal nerve, enhances neural connectivity within the salience network and diminishes the salience-to-DMN connectivity. This research marks an initial step toward exploring olfactory nerve stimulation as a potential therapeutic approach for treatment-resistant depression. As the first study of its kind, we investigated these effects in healthy individuals.

## Methods

### Participants

We recruited 45 *healthy individuals* with a mean age of 29.6 years (*SD* = 12.8, range = 19–64 years of age) through advertisement at the University Clinic Carl Gustav Carus in Dresden, Germany (see Supplementary Material for power calculation). Following the skewed gender proportion in depression, we investigated 15 male and 30 female participants. Exclusion criteria were mental disorders as screened with the Patient Health Questionnaire (PHQ-D) [32] and disorders related to loss of olfactory function (e.g. Parkinson’s disease, renal insufficiency, inflammatory diseases in the nose) or neurodevelopmental disorders. To maximize the potential effect of olfactory nerve fiber stimulation, we furthermore excluded dysosmic individuals, as screened by the Sniffin’ Sticks identification test (Burghart Messtechnik GmbH, Germany) with the cut off of 12/16 [33].

The study was approved by the ethics committee of the Technical University Dresden (application number EK417092019) and performed under the ethical guidelines of the Helsinki Declaration [34]. All participants provided written informed consent. They received moderate financial compensation.

### Research design

#### Procedure

**Fig. 1:**
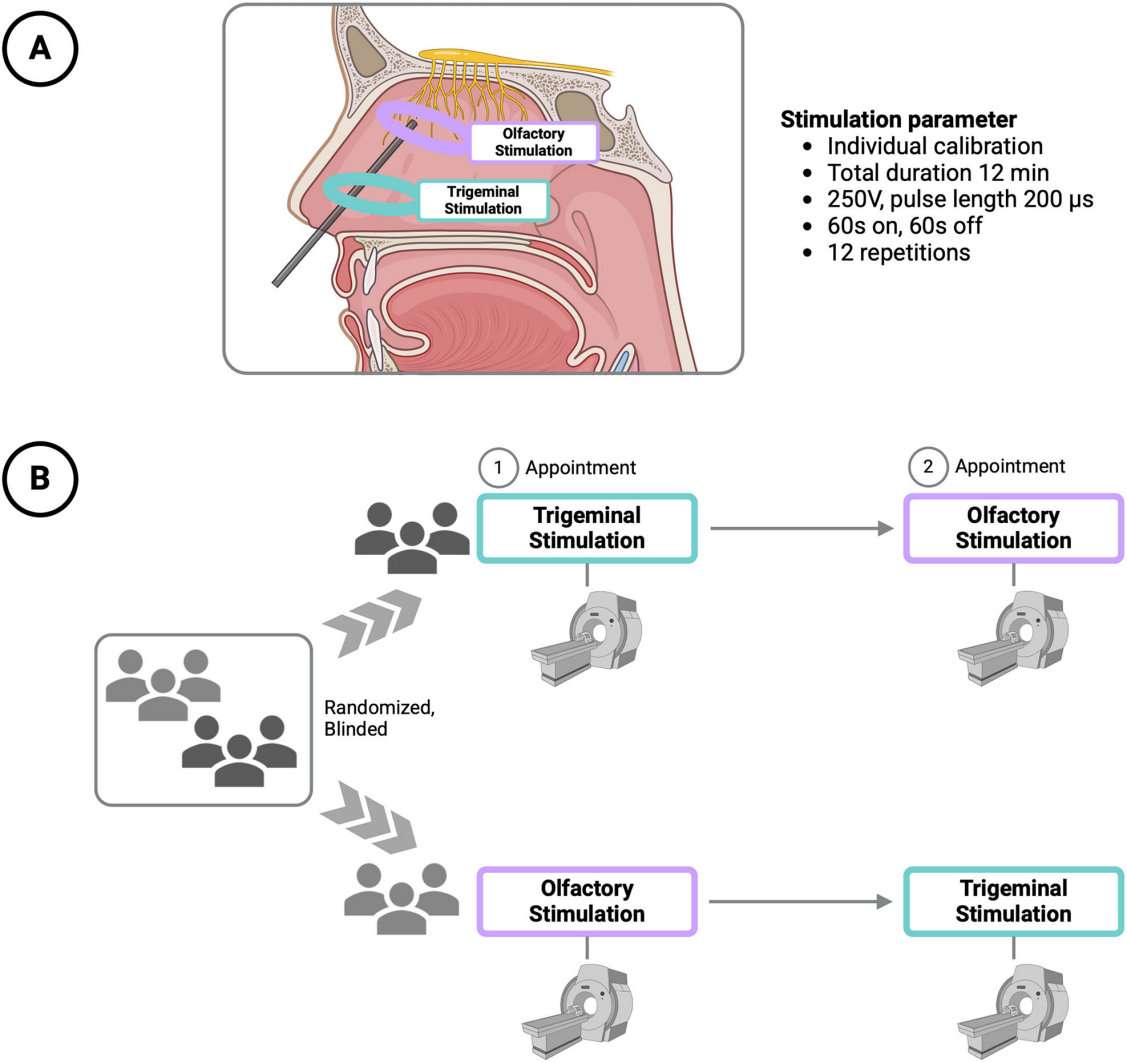
Study design and procedure. **A** shows the sagittal cross-section of the nasal cavity. The positioning of the electrode head in the olfactory stimulation condition is highlighted in purple. The positioning of the electrode head for the trigeminal stimulation condition is highlighted in turquois. **B** shows the schematic representation of the timeline of the study. Participants attended two randomized experimental sessions. In each session, they received either olfactory or trigeminal nerve stimulation, followed by an MRI scan. Created with Biorender.com.

Following an initial session to assess normosmia and calibration of electrical stimulation based in individual thresholds, participants attended two randomized experimental sessions. In each session, they received either olfactory or trigeminal nerve stimulation, followed by MRI scans (see Fig. 1). To minimize the delay between end of stimulation and start of MRI assessment, electrical stimulations were conducted in a room adjacent to the MRI scanner, replicating the successful protocol established by Weiss and colleagues [31].

To monitor potential associated phenomena of the stimulation, participants rated the intensity of sensations “tingling”, “stabbing”, “burning”, and “cooling” (based on Weiss et al. [31]) on a 5-point Likert scale (ranging from no sensation to very strong) after the MRI session. Based on our previous experience with such stimulation, we also asked participants to assess further potential associated phenomena, namely “toothache”, “headache”, “numbness” and “paresthesia” on a 5-point Likert scale (ranging from none to very strong). Furthermore, we encouraged them to report any further sensations.

To monitor potential changes in implicit affect resulting from electrical stimulation, participants completed the Implicit Positive and Negative Affect Test (IPANAT) [35] before and after both the olfactory and trigeminal stimulation.

#### Electrical stimulation

The major difference between the olfactory and trigeminal conditions was in the intranasal electrode placement (see Fig. 1). For the olfactory condition, electrodes were positioned on the upper part of the middle nasal turbinate close to its insertion at the lateral wall, following the methodology of Weiss et al. [31], whereas for the trigeminal condition, electrodes were placed on the lower nasal turbinate, where trigeminal sensitivity to electrical stimulation is typically higher [36]. To ensure stimulation specificity, the lower nasal turbinate was selected for the trigeminal condition because it lies outside the known distribution of olfactory sensory neurons. While the olfactory epithelium can reach from the olfactory cleft up to the middle turbinate, especially in younger individuals [37, 38], there is no anatomical or histological evidence supporting the presence of olfactory receptors in the lower turbinate.

Small spherical electrodes (6 mm; COP06S1-80, SEI EMG s.r.l., Cittadella IT) were applied to the designated locations under endoscopic guidance. Electrical impulses were generated and controlled using a high-voltage direct current stimulator (DSA7, Digitimer, UK) and monitored with an oscilloscope (GoldStar Oscilloscope OS-9020G; Rigol, Suzhou, China). To conceal the stimulation condition from participants, both electrodes, olfactory and trigeminal, were attached in each condition and the position was stabilized during stimulation using a glasses-like frame.

For the electrical stimulation, participants were instructed to lie quietly with their eyes closed on a stretcher. Stimulation was administered for 12 min at a voltage of 250V, with an on-time of 60 s, and an off-time of 60 s. During on-time stimulation blocks occurred in 6 on-time cycles, 200 µs pulse width, 10 Hz square wave.

These parameters were adapted from Weiss et al. [31] and refined based on a pre-study involving 16 participants (see Supplementary Material). To ensure perceptual consistency across participants and conditions, individual calibration was performed in the initial session. Therefore, we determined the intranasal perception thresholds for both olfactory and trigeminal stimulation by gradually increasing the current of single impulses by 0.05 milliamperes (mA) starting from 0.5 mA, with a pulse duration of 500 μs until participants reported to perceive the stimulus. During the experimental sessions, stimulation was delivered at twice the perception threshold (average over all participants; olfactory, threshold: *M* = 1.50 mA, *SD* = 0.401 mA; stimulation: *M* = 3.00 mA, *SD* = 0.801 mA, range = 0.75–2.65 mA; trigeminal, threshold: *M* = 0.917 mA, *SD* = 0.278 mA; stimulation: *M* = 1.83 mA, *SD* = 0.556 mA, range = 0.50–1.65 mA).

#### Image acquisition

Imaging data were acquired using a 3T MAGNETOM Prisma scanner (Siemens Healthcare, Erlangen, Germany) equipped with a 64 channel head coil. In two cases, a 20-channel head coil was used due to the large head size and the need for MRI compatible glasses which did not fit the 64 channel head coil.

First, a T1-weighted anatomical image was obtained with a magnetization prepared – rapid gradient echo (MPRAGE) sequence (volumes: 1, axial slices: 160, slice thickness: 1.0 mm, FoV: 256 mm, TR: 2300 ms, TE: 3.43 ms, TA: 5 min 12 s, flip angle: 9°, voxel size: 1.0 × 1.0 × 1.0 mm^3^). This was followed by a resting state scan (volumes: 250, axial slices: 35, slice thickness: 2.2 mm, FoV: 220 mm, TR: 2100 ms, TE: 30.0 ms, TA: 8 min 53 s, flip angle: 90°, voxel size: 2.2 × 2.2 × 2.2 mm^3^), during which participants were instructed to close their eyes. Lastly, a task-based sequence was acquired where the participants performed a salience evoking Oddball-paradigm [13]. This task is not within the scope of the current study and is presented in full in the Supplementary Information for reasons of transparency [13].

### Statistical analysis

#### Analysis of associated phenomena and sensations

Potential associated phenomena and sensations were descriptively analyzed. Furthermore, an aggregated total score was computed by summing the ratings of phenomena per participant. This was done separately for both the olfactory and trigeminal stimulation. Wilcoxon singed-rank tests (two-tailed) were performed in R (https://www.r-project.org) to analyze the statistical difference from zero and between the two conditions, both for the individual phenomena and for the total score. Identically, an aggregated intensity score for all perceived sensations during the stimulation was calculated and Wilcoxon singed-rank tests (two-tailed) were performed.

#### Resting-state functional MRI data

Preprocessing was conducted using the CONN toolbox preprocessing pipeline including slice time correction and smoothing with a 7 mm FWHM Gaussian kernel [39]. Subsequent denoising steps involved the regression of five principal components from white matter, five from cerebrospinal fluid, 12 from realignment, and 38 from scrubbing, along with linear detrending. To further minimize noise related to cardiac and respiratory activity, a temporal band-pass filter of 0.01–0.1 Hz was applied to the time series as suggested by previous research [40, 41].

**Fig. 2:**
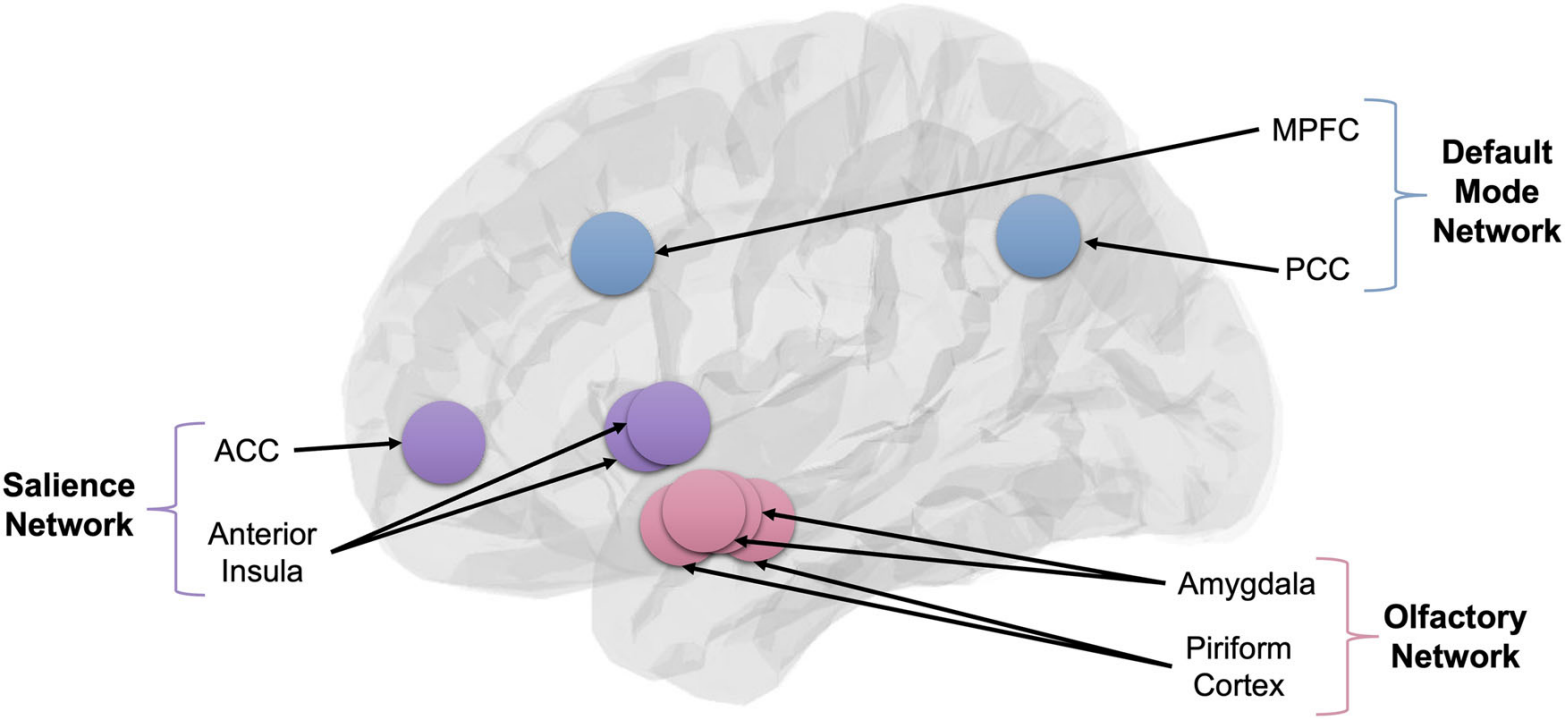
Visualization of the salience, default, and olfactory network. ACC anterior cingulate cortex, MPFC medial prefrontal cortex, PCC posterior cingulate cortex.

We focused analysis on three key networks: the salience network (including the left and right anterior insula and the ACC), the DMN (including the posterior cingulate cortex (PCC) and the MPFC), both based on own prior investigations [11, 14], and the olfactory network (including the left and right amygdala and the left and right piriform cortex). ROI definitions were based on anatomical locations provided in the CONN toolbox, except for the piriform cortex which was manually defined following the recommendations of Thaploo and colleagues [42]. The ROIs are visualized in Fig. 2.

First-level ROI-to-ROI analysis was performed on the preprocessed data. Second level ROI-to-ROI analysis was calculated separately for the olfactory and the trigeminal condition, as well as for between-condition contrasts (olfactory > trigeminal; olfactory < trigeminal). Contrast results and effect sizes were visualized and extracted using CONN’s results explorer tool. Two key measures were used to describe networks of strongly connected ROIs: *Network Size* (the number of strong connections) and *Network Mass* (the strength of those connections, calculated as the sum of statistical values [T-squared] across all connections in a given network). To control for multiple comparisons, we applied the false discovery rate (FDR) correction at the network level, using a significance threshold of *p* < 0.05. For the initial thresholding of the ROI-to-ROI matrix however, an uncorrected *p*-value of *<*0.05 was applied to reduce the risk of type II errors in this initial study.

To further assess how different stimulations influenced connectivity within and between the key brain networks, individual estimates were extracted from CONN for each participant, connection, and condition and entered in repeated measurement ANOVAs using JASP (version 0.18.3.0). The within subject effects of condition (2) and connection were modeled as main and interaction effects, with Greenhouse Geiser correction applied to account for sphericity violations. The analyses were conducted both within each network (salience [6 connections], default-mode [2 connections], olfactory [10 connections]), as well as for salience- default mode [8 connections] and salience-olfaction [18 connections] network connectivity.

#### Analysis of implicit mood

Wilcoxon singed-rank tests (two-tailed) were performed to examine whether total scores for positive and negative implicit affect differed between pre- and post-stimulation within each condition. Analyses were performed in R (https://www.r-project.org), separately for positive and negative affect, and for olfactory and trigeminal stimulation.

## Results

### Evaluation of associated phenomena and sensations for the olfactory and trigeminal stimulation

**Fig. 3:**
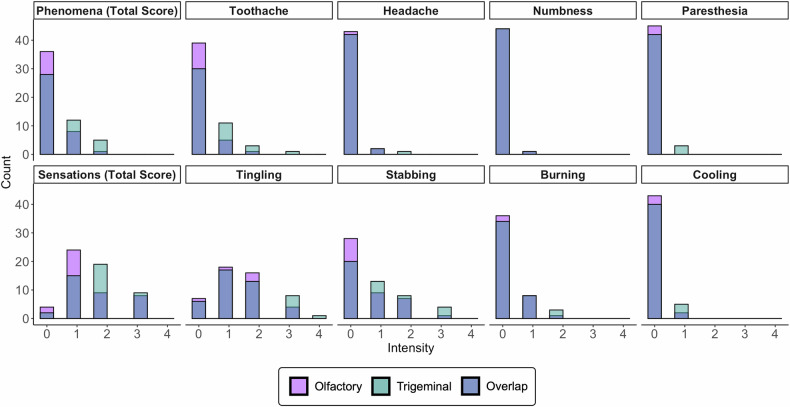
Density histogram displaying the distribution of associated phenomena and sensations caused by olfactory and trigeminal stimulation. During the electrical stimulation and after the MRI session, participants were asked to rate both the olfactory and the trigeminal stimulation for the intensities of the associated phenomena (toothache, headache, numbness and paresthesia) and the intensities of the sensation (tingling, stabbing, burning, cooling). They rated the intensities of each phenomenon and sensation on a 5-point Likert scale ranging from 0 (no sensation) to 4 (very strong). An aggregated total score for phenomenon intensity was computed by summing the ratings of each phenomenon for each participant, for both the olfactory and trigeminal stimulation respectively. The same procedure was repeated for the total score for sensation intensity. The x-axis represents the intensity of associated phenomena and sensations. The y-axis represents the count. Purple olfactory stimulation, Turquoise trigeminal stimulation, Blue overlap between olfactory and trigeminal stimulation.

Participants rated associated phenomena and sensations significantly above zero (all *p* < 0.005), but with very low intensity. The intensity was higher during trigeminal than during olfactory stimulation (associated phenomena (total score): *p* = 0.0036; sensations (total score): *p* = 0.0386; see Fig. 3).

Looking at individual items, toothache was the only associated phenomena that significantly deviated from zero. This was true both in the olfactory (*p* = 0.0263) and the trigeminal condition (*p* < 0.0004), but stronger in the trigeminal one (*p* = 0.0016). Additionally, one participant reported an associated phenomenon of “a pulling feeling in the nasal cavity” (intensity = 1), one a “throbbing in the hand” (intensity = 1), and one a “pressure” (intensity = 1) during the olfactory condition. During the trigeminal condition, one participant reported throbbing in the tooth (intensity = 2), and another reported pressure in the head (intensity = 2). For sensations, intensities for stabbing, tingling, and burning deviated significantly from zero for the olfactory and trigeminal condition (all *p* < 0.005) and cooling was significantly reported in the trigeminal condition only (*p* = 0.0369). The sensation of stabbing was significantly stronger for the trigeminal than for the olfactory condition (*p* = 0.0333), for all other sensations, there was no significant group difference (see Fig. 3). Additionally, six participants reported sensations described as throbbing (intensities = 1, 1, 2, 3, 3, 3), one as pulsing (intensity = 2), one as pressure (intensity = 1), one tapping in wrist (intensity = 1) during the olfactory condition, while four participants reported throbbing (intensities = 1, 1, 2, 2), one tapping (intensity = 1), one pulsing (intensity = 3), and one pressure (intensity = 1) during the trigeminal condition. One participant reported the smell of leaves after the olfactory stimulation. No further experiences of smell were reported.

### Connectivity within and between networks

**Fig. 4:**
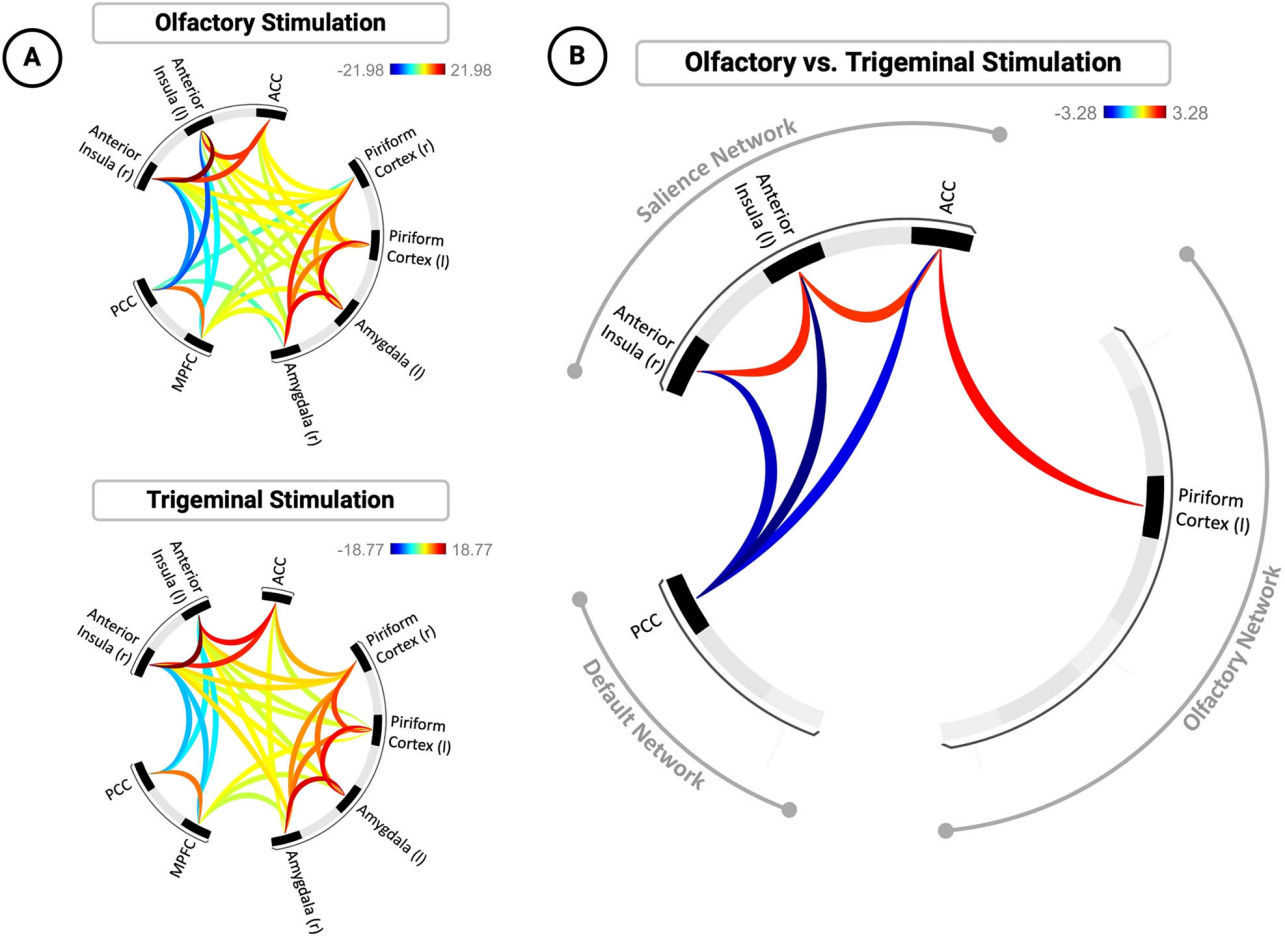
Resting-state functional connectivity within and between networks. **A** Functional connectivity during olfactory and trigeminal stimulation separately. **B** Functional connectivity compared between conditions (olfactory vs. trigeminal stimulation). Red lines indicate increased connectivity within networks, while blue lines indicate decreased connectivity between networks. r right hemisphere, l left hemisphere, ACC anterior cingulate cortex, MPFC medial prefrontal cortex, PCC posterior cingulate cortex.

After both, olfactory and trigeminal stimulation, we observed pronounced functional connectivity within regions of the salience network, DMN, and the olfactory network (olfactory: mass value = 5595.42, *p*_FDR-corrected_ < 0.001; trigeminal: mass value = 4241.33, *p*_FDR-corrected_ < 0.001; see Fig. 4A). After both stimulations, the most pronounced connectivity was observed within the salience network, particularly between the left and right anterior insula (olfactory: *T*[44] = 21.98, *p*_FDR-corrected_ < 0.001; trigeminal: *T*[44] = 18.77, *p*_FDR-corrected_ < 0.001). Moreover, both stimulations showed significantly negative functional connectivity between the salience network and DMN. This negative functional connectivity was most pronounced between the PCC of the DMN and the left anterior insula of the salience network (olfactory: *T*[44] = −12.80, *p*_FDR-corrected_ < 0.001; trigeminal: *T*[44] = −7.21, *p*_FDR-corrected_ < 0.001).

Comparing olfactory and trigeminal stimulation conditions revealed significant differences in functional connectivity patterns (overall connectivity: mass value = 82.51, *p*_FDR-corrected_ = 0.026, see Fig. 4B). Specifically, olfactory compared to trigeminal stimulation increased functional connectivity within the salience network and between the salience network and the piriform cortex of the olfactory network. Furthermore, olfactory stimulation decreased functional connectivity between the PCC of the DMN and all regions of the salience network (see Table 1).

**Table 1:** Functional connectivity results of individual connections (Olfactory vs. Trigeminal Stimulation).

Analysis Unit	Statistics	*p*-unc	*p*-FDR
**Networks (Salience, DMN, Olfactory)**	Mass = 82.51	0.026	0.026
PCC – Right Anterior Insula	*T*(44) = −3.28	0.002	0.073
PCC – Left Anterior Insula	*T*(44) = −2.86	0.006	0.115
PCC – ACC	*T*(44) = −2.64	0.011	0.136
Piriform Cortex – ACC	*T*(44) = 2.40	0.021	0.186
Right Anterior Insula – Left Anterior Insula	*T*(44) = 2.23	0.031	0.222
ACC – Right Anterior Insula	*T*(44) = 2.14	0.038	0.228

*DMN* default mode network, *ACC* anterior cingulate cortex, *PCC* posterior cingulate cortex, *p-unc* uncorrected *p*-value, *p-FDR* false discovery rate-corrected *p*-value.

**Fig. 5:**
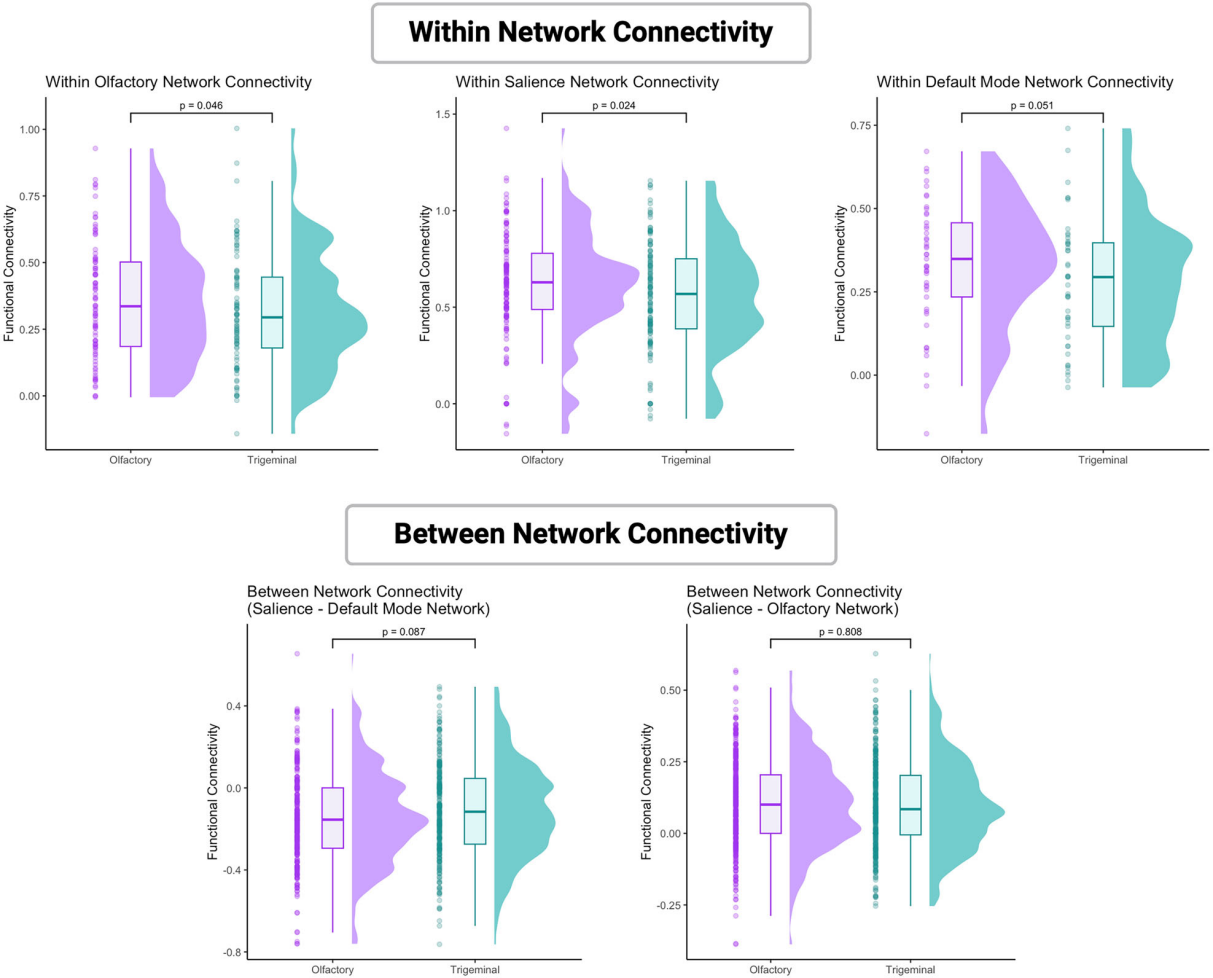
Differences in functional connectivity between the olfactory and the trigeminal stimulation. The plot combines a boxplot (center) representing the median and interquartile range, a violin plot (cloud, right) illustrating the density distribution of the data, and individual data points (rain, left) overlaid for clarity. This visualization highlights both the central tendency and variability of the data, as well as the individual observations within each condition.

Analysis of extracted data allowed for combined analysis of all network areas and unsurprisingly confirmed the increased connectivity after the olfactory vs trigeminal stimulation within the hubs of the salience network (*F*[1,43] = 5.5, *p* = 0.024, η^2^ = 0.113). The analysis also showed a significantly increased connectivity within the bilateral core hubs of the olfactory network (*F*[1,43] = 4.2, *p* = 0.046, η^2^ = 0.089) during olfactory stimulation as opposed to the trigeminal stimulation. In tendency, the same pattern was observed in the default mode network, but missed statistical significane (*F*[1,43] = 4.0, *p* = 0.051, η^2^ = 0.086). Between-network connectivity did not differ between olfactory and trigeminal stimulation, neither for the salience-olfactory network (*F*[1,43] = 0.1, *p* = 0.808, η^2^ = 0.001), nor for the salience-default mode network (*F*[1,43] = 3.1, *p* = 0.087, η^2^ = 0.066). However, the latter between-network connectivity was in tendency reduced during olfactory stimulation (see Fig. 5).

### Evaluation of implicit mood

**Fig. 6:**
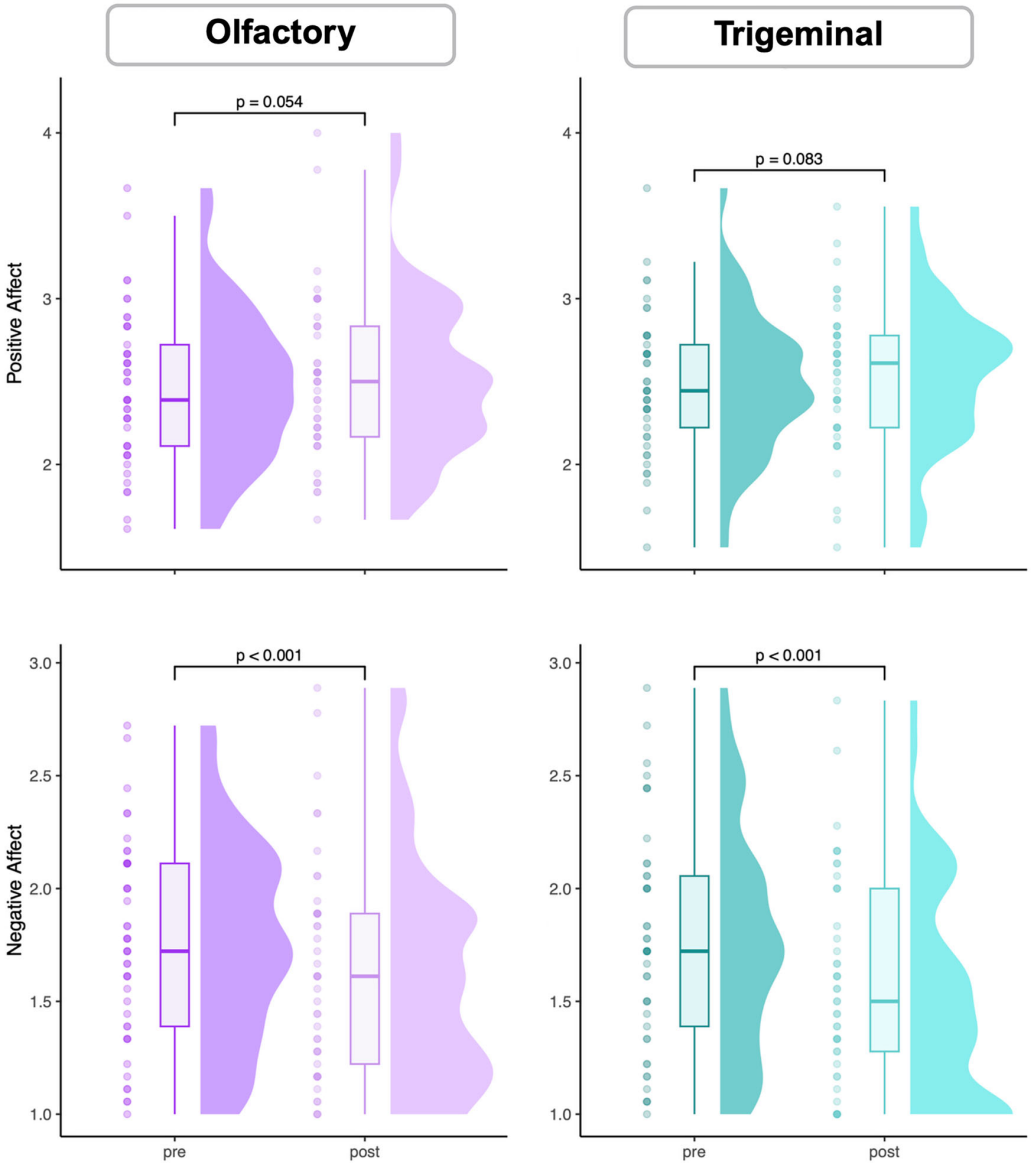
Differences in implicit positive and negative affect pre- and post-stimulation in both the olfactory and the trigeminal condition. The plot combines a boxplot (center) representing the median and interquartile range, a violin plot (cloud, right) illustrating the density distribution of the data, and individual data points (rain, left) overlaid for clarity. This visualization highlights both the central tendency and variability of the data, as well as the individual observations pre- and post-stimulation within each condition.

Implicit positive affect did not significantly differ between pre- and post-stimulation in either condition (olfactory: *p* = 0.054; trigeminal: *p* = 0.083). In contrast, implicit negative affect showed a significant reduction following stimulation in both conditions (olfactory: *p* < 0.001; trigeminal: *p* < 0.001), suggesting a decrease in negative affect post-stimulation regardless of condition (see Fig. 6).

## Discussion

This study investigated the effects of electrical stimulation of the olfactory mucosa on salience network connectivity in healthy individuals. In line with the olfactory processing pathway, olfactory nerve stimulation significantly increased functional connectivity within the olfactory cortex, between the olfactory and the salience network, and within the salience network regions. Furthermore, olfactory nerve stimulation decreased functional connectivity between the salience network and the PCC of the DMN.

The increased olfactory cortex connectivity indicates that electrical stimulation of the olfactory area leads to synchronization of activation patterns in the piriform cortex and the amygdala – a result similarly observed by Weiss and colleguages [31]. The increased connectivity between the olfactory and the salience network furthermore suggests that electrical stimulation can influence brain activity of the salience network through olfactory processing pathways. Notably, functional connectivity between the piriform cortex and the salience network was also observed in the trigeminal stimulation condition. This may reflect a baseline level of connectivity between the piriform cortex and salience-related structures (as indicated by structural connectivity [43]). However, this functional connectivity was significantly stronger in the olfactory condition. Given the complexity of innervation in the nasal cavity [44, 45], we cannot completely rule out the possibility that trigeminal stimulation may have inadvertently engaged some olfactory nerve fibers. It is, however, highly unplausible that olfactory nerve fibers were activated in the trigeminal condition.

In the context of depression, it is particularly noteworthy that olfactory stimulation increased connectivity within the salience network and while decreasing connectivity between the salience and the DMN. The is striking because the opposite pattern, namely reduced functional connectivity within the salience network [8] alongside increased connectivity between the salience network and the DMN [10, 46, 47] is typical for depression. Additionally, connectivity within the salience network is inversely associated with the severity of depression symptoms [10], while increased DMN influence on the salience network has been linked to negative cognitive biases in processing positive stimuli [48]. Furthermore, reduced functional connectivity of the right insula within the salience network has been suggested as a marker for poor antidepressant response [49]. These findings raise the question of whether modulating resting-state functional connectivity through olfactory nerve stimulation could benefit individuals with depression. The functional changes observed in our study suggest that olfactory stimulation may counteract some psychopathological alterations, offering a potential minimal invasive neuromodulatory approach in the treatment of depression.

Our study was conducted with healthy individuals to test for effects, safety, and associated phenomena and sensations of olfactory nerve fiber stimulation. We did not observe significant differences in positive affect between pre- and post-stimulation in either condition. Negative affect significantly decreased following both stimulations, potentially reflecting reduced tension after ending the somewhat distressing experimental procedure. However, it should be noted that both positive and negative affect were generally low and within non-pathological ranges, as the study was performed in a health participant sample. Future studies should investigate the antidepressant potential of such stimulation in patients with depression, and analyze how its efficacy compares to established treatments, such as pharmacotherapy and auricular vagus nerve stimulation [50–53], both known to alter resting state network connectivity [53]. We do not yet know if olfactory stimulation is another promising intervention for depression treatment. While it shows a favorable associated phenomena profile in healthy participants, we have no data on its efficacy and phenomena — particularly regarding long-term associated phenomena in patients with depression.

Few participants reported toothache following stimulation, with slightly more reports after trigeminal than olfactory stimulation. To mitigate such symptoms and preserve patient compliance in clinical applications, reducing the electrical amplitude could be a feasible strategy. However, this must be carefully balanced against the potential risk of diminishing the desired neural engagement. Replicating our findings in clinical samples will provide critical insights into whether olfactory stimulation can serve as an effective and safer alternative to more invasive treatments, offering new avenues for individualized depression therapy with potentially fewer side effects.

### Limitations

Some limitations must be noted. First, the generalizability of our findings is restricted due to the sample, that predominantly consists of younger, well-educated individuals. Individuals with treatment resistant depression are on average a little older, and less educated than our participants were [54]. Given that functional connectivity within and between brain networks change with age [55], these age-related changes could potentially alter the effects of olfactory stimulation in older individuals, which should be considered when interpreting the results. Second, the study sample presents an intended sex imbalance, with twice as many females (*n* = 30) as males (*n* = 15), reflecting the higher prevalence of depression in females. However, the smaller number of male participants hindered us to perform sex-specific analyses due to the increased risk of false positive or false negative results. Future studies with larger sample sizes and more balanced sex representation would help elucidate whether sex differences exist in salience network modulation due to olfactory stimulation and, in turn, provide insights into the sex-specific neural underpinnings of depression. Third, the stimulation effects were measured using resting-state fMRI taken approximately eight minutes after the electrical stimulation, which only allows for the assessment of short-term effects. The duration and persistence of the observed effects remain unclear. Additionally, the stimulation paradigm was novel, with parameters selected in a pilot-study to minimize side effects while ensuring effective stimulation. It is uncertain how variations in stimulus intensity might influence the results, and the potential impact of different stimulation thresholds on the observed changes in functional connectivity should be carefully considered in future research.

## Conclusion

This study highlights the potential of olfactory nerve stimulation to modulate functional connectivity within the brain’s salience network, with observed increased connectivity within the salience network and decreased connectivity between the salience network and the DMN. The findings suggest that olfactory stimulation could influence brain activity in ways that contrast those observed in depression, potentially offering a new avenue for therapeutic intervention. Given that these results were observed in a healthy cohort, further research is needed to determine their applicability in clinical settings.

## Supplementary information

Supplementary Material

## Data Availability

Data is available from the corresponding author on request.
