# Supplementary material for: Modulating salience network connectivity through olfactory nerve stimulation
Source: Transl Psychiatry. 2025 Aug 21;15:303. doi: 10.1038/s41398-025-03500-6 (PMC12370952; doi:10.1038/s41398-025-03500-6)
Supplement: Supplementary file 1 — Supplementary Material [file 41398_2025_3500_MOESM1_ESM.docx]

**Supplementary Material**

**Modulating Salience Network Connectivity through Olfactory Nerve Stimulation**

Carina Heller, Maria Geisler, Nicolas Mayer, Annabelle Thierfelder, Martin Walter, Thomas Hummel,

Ilona Croy

[Sample size and power calculation 2](#_Toc198987083)

[Determination of stimulation of the olfactory and trigeminal nerve in pilot study 2](#_Toc198987084)

[Functional MRI Paradigm in Main Study 5](#_Toc198987085)

[Preprocessing of task-based functional MRI in Main Study 6](#_Toc198987086)

[Statistical analysis of task-based functional MRI data in Main Study 6](#_Toc198987087)

[Results of task-based functional MRI data in Main Study 7](#_Toc198987088)

[References 9](#_Toc198987089)

# *Sample size and power calculation*

There are currently no universally accepted guidelines for determining optimal sample sizes in fMRI studies investigating affective touch. However, based on our prior experience, key regions of interest (amygdala, anterior cingulate cortex, and anterior insula) can be reliably detected with a sample size of approximately 20 participants. To ensure sufficient power for within-subject comparisons across two conditions, we aimed to recruit 2 × 20 = 40 participants. To account for potential dropouts or technical issues associated with this demanding paradigm, we included a buffer, resulting in a total sample size of 45 participants.

# *Determination of stimulation of the olfactory and trigeminal nerve in pilot study*

Guided by previous literature^1^, the aim of the pilot study was to target deep brain networks by olfactory stimulation. In contrast to Weiss et al., our target was the salience network. Hence, we optimized the stimulation parameters by Weiss et al. and used an oddball salience-evoking oddball paradigm. Determining the stimulation sequence for the electrical intranasal stimulation, 16 healthy and normosmic participants (10 female) with a mean age of 27.25 years (*SD* = 10.74 years, range = 19 – 59 years of age) were acquired through advertisement of the study at the University Clinic in Dresden, Germany. Normosmia was tested using the standardized Sniffin’ Sticks odor identification test (Burghart Messtechnik GmbH, Germany) with the cut off 12 of 16^2^. The absence of mental health impairments was identified with the Patient-Health-Questionnaire (PHQ-D)^3^. The intranasal electric threshold was specified individually for each participant using small spherical electrodes (COP06S1-80, SEI EMG s.r.l., Cittadella IT) which were positioned on the upper part of the middle nasal turbinate close to its insertion at the lateral wall, following the methodology of Weiss et al.^1^, whereas for the trigeminal condition, electrodes were placed on the lower nasal turbinate, where trigeminal sensitivity to electrical stimulation is typically higher^4^. The second position “trigeminal” was in the lower area of the nasal septum which is innervated by the trigeminal nerve^5^. The electrodes were fixated on a clip mounted to lensless glasses worn by the person throughout threshold determination and stimulation. The electrodes were connected to a stimulator generating the electrical current (DSA7, Digitimer, UK). Starting at 0.5 mA, 250V and a stimulus duration of 500 µs the amperage was increased in 0.05 mA steps until the participant reported to perceive the single electrical impulse^6,7^. For the stimulation the stimulator was also connected to an oscilloscope (GoldStar Oscilloscope OS-9020G; Rigol, Suzhou, China) to create continuous small electrical shocks throughout the on-time (frequency = 10 Hz, function = square wave) while different stimulation sequences were conducted through the olfactory and trigeminal electrode in the nose (see Table 1). The stimulation was performed at twice the amperage of the individual threshold (average over all participants; olfactory, threshold: M = 1.025 mA, SD = 0.457 mA; stimulation: M = 2.019 mA, SD = 0.952 mA, range = 0.15 – 2.00 mA; trigeminal, threshold: M = 1.109 mA, SD = 0.282 mA; stimulation: M = 2.156 mA, SD = 0.598 mA, range = 0.55 – 1.70 mA). During stimulation, a laptop presenting a salience-evoking oddball paradigm was placed in front of the participants. They were asked to react to every smiling face in the paradigm by pressing the button “*J”* on the keyboard. The reaction time to positive stimuli, percentage of hits *[(hits / (hits + misses)]* and accuracy *[(hits + correct rejections) / (false alarms + hits + misses + correct rejections)]* was calculated from the paradigm data. The results suggest that a stimulation sequence with a pulse length of 200 µs, an on-time of 60 s and an ISI of 60 s is the most efficient way for reacting to a salience-evoking oddball paradigm (see Table 2).

| Table 1. Stimulation Patterns and Results of Salience-Evoking Oddball Paradigm per Participant | | | | | | | | | |
| --- | --- | --- | --- | --- | --- | --- | --- | --- | --- |
| Participants | **Stimulation**  **Sequence** | **Stimulation Area** | **Pulse Length** | **On-Time** | **InterstimulusInterval (ISI)** | **Reaction Time**  **(*M*±*SD*)** | **Hits**  **(*M*±*SD*)** | **Accuracy**  **(*M*±*SD*)** |  |
| 1 - 8 | 1 | olfactory | 100 µs | 120 s | 30 s | 721.11± 27.93 ms | 0.79 ± 0.043 | 0.90 ± 0.014 |  |
|  | 2 | olfactory | 500 µs | 60 s | 90 s | 695.10 ± 27.85 ms | 0.78 ± 0.044 | 0.92 ± 0.014 |  |
|  | 3 | olfactory | 100 µs | 60 s | 60 s | 683.48 ± 44.31 ms | 0.78 ± 0.037 | 0.93 ± 0.013 |  |
|  | 4 | trigeminal | 500 µs | 120 s | 30 s | 719.42 ± 33.22 ms | 0.80 ± 0.038 | 0.92 ± 0.011 |  |
| 9 - 16 | 5 | olfactory | 200 µs | 15 s | 30 s | 661.51 ± 26.29 ms | 0.79 ± 0.025 | 0.91 ± 0.012 |  |
|  | 6 | olfactory | 200 µs | 120 s | 60 s | 668.33 ± 14.78 ms | 0.73 ± 0.040 | 0.91 ± 0.012 |  |
|  | 7 | olfactory | 500 µs | 15 s | 90 s | 677.23 ± 26.79 ms | 0.77 ± 0.026 | 0.91 ± 0.008 |  |
|  | 8 | trigeminal | 500 µs | 120 s | 30 s | 695.75 ± 14.48 ms | 0.77 ± 0.036 | 0.91 ± 0.018 |  |

| Table 2. Mean Results of Salience-Evoking Oddball Paradigm | | | | | | |
| --- | --- | --- | --- | --- | --- | --- |
|  |  |  | **Mean Accuracy** | **SD**  **Accuracy** | **Mean**  **Reaction Time** | **SD**  **Reaction Time** |
| Pulse Length  On Time  Interstimulus Interval (ISI) |  | 100 µs | 0.9126 | 0.0406 | 702.3 | 106.4 |
|  |  | 200 µs | 0.9109 | 0.0350 | 664.9 | 60.4 |
|  |  | 500 µs | 0.9187 | 0.0330 | 686.2 | 77.8 |
|  |  | 15 s | 0.9116 | 0.0292 | 669.4 | 75.5 |
|  |  | 60 s | 0.9254 | 0.03847 | 689.3 | 104.8 |
|  |  | 120 s | 0.9052 | 0.03810 | 694.7 | 68.5 |
|  |  | 30 s | 0.9041 | 0.03800 | 691.3 | 82.3 |
|  |  | 60 s | 0.9194 | 0.03628 | 675.9 | 93.7 |
|  |  | 90 s | 0.9187 | 0.03299 | 686.2 | 77.8 |

Note. The results suggest that a stimulation sequence with a pulse length of 200 µs, an on-time of 60 s and an ISI of 60 s (marked in green) is the most efficient way for reacting to a salience-evoking oddball paradigm.

# *Functional MRI Paradigm in Main Study*

Based on a previous publication^8^, we amended a social Oddball paradigm to selectively evoke neural activity in the salience network ^9–12^. Subtly smiling faces were chosen as target stimuli, embedded in a sequence of faces with neutral expressions. These stimuli were presented to the participant lying in the MRI scanner on a television screen. Stimuli were presented 1500 ms, followed by a shorter interstimulus interval (ISI) of 500 ms, during which a fixation cross was displayed. In response to the appearance of subtly smiling faces, participants were instructed to press a button with their right thumb. Data from another study using an Oddball paradigm suggest that the chosen presentation time of 1500 ms for a button press is sufficient^13^. If the button press was correctly executed upon the presentation of a smiling face, the feedback “Great!” appeared instead of the fixation cross. If the face was not recognized as smiling and the button was not pressed, the feedback "Wrong!" was displayed. A minimum time interval of 14,000 ms between presentation of smiling faces ensured that the BOLD signal normalizes after rise^10^. Varying the temporal difference from 14,000 ms to 18,000 ms between runs resulted in the presentation of a positive, smiling face as the target stimulus after seven, eight, or nine neutral faces. With a total of 176 faces and a duration of the paradigm of approximately 6 minutes and 30 seconds, this corresponds to a percentage of stimuli of 12% (21 faces) and a percentage of neutral faces of 88% (155 faces). The presentation order of faces in the paradigm was randomized for each participant and each run to minimize habituation and learning effects. The paradigm was presented using Presentation® Software (Version 18.0, Neurobehavioral Systems, Inc., Berkeley, CA, [www.neurobs.com](http://www.neurobs.com)). The 48 neutral faces and subtly smiling stimuli used in total, which served as the basis for creating the paradigm, were obtained from previous literature^14^ and generously made available by the research group of Prof. Dr. Stefan R. Schweinberger.

# *Preprocessing of task-based functional MRI in Main Study*

Analysis of the fMRI data (volumes: 248, axial slices: 24, slice thickness: 2.5 mm, FoV: 220 mm, TR: 1510 ms, TE: 30.0 ms, TA: 6 min 19 s, flip angle: 90°, voxel size: 2.5x2.5x2.5 mm^3^) was performed using SPM12 (http://www. fil.ion.ucl.ac.uk/spm) ^15^ implemented in Matlab R2017b (MathWorks Inc., Natick, MA, USA). DICOM files were imported to NifTI-format by the conversion utility implemented in SPM12. For the preprocessing, we used the default settings implemented in SPM 12. After slice time correction (reference slice = last slice), functional images were realigned to the first image of the functional run using a 2nd degree B-spline and unwarped with a 4th degree B-spline. This was followed by co-registration of the T1-weighted image with the averaged functional mean image and segmentation fitting (bias regularization = 0.0001, bias FWHM = 60 mm cutoff). The realigned images were normalized into MNI ICBM 152-space using 4^th^ degree B-Spline interpolation (voxel size = 2.4x2.4x2.4). Thereafter, we smoothed the normalized images using a FHWM Gaussian kernel of 7 mm.

# *Statistical analysis of task-based functional MRI data in Main Study*

First level analysis was performed event-related based on the stimulus onsets, taken from the presentation software logfile data and implemented in MATLAB. We first contrasted hits vs correct rejection, for each run (olfactory vs trigeminal) separately using SPM 12. For the SPM 12 second-level analyses, we calculated T-tests thresholded at *p*_FWE_ <.05: hit vs. correct rejection (for both olfactory and trigeminal stimulation). This was done over all conditions and separately for the olfactory and the trigeminal condition. Thereafter, we run a small volume correction for each ROI respectively and extracted the peak beta signals within each ROI and subject for both conditions (olfactory and trigeminal stimulation).

The extracted signals were entered in a repeated measurement ANOVA running under JASP. Condition, event (hit vs correct rejection) and each of the ROIs served as within-subject condition. Greenhouse Geiser correction was implemented due to violation of sphericity, the main effects of condition and event were modelled and the condition by ROI interaction.

# *Results of task-based functional MRI data in Main Study*

In order to determine the validity of the oddball-paradigm, we first tested, whether participants reacted correctly to the stimuli. They were able to correctly react to the stimuli with an accuracy of 88.3% in the olfactory condition and 86.4% in the trigeminal condition. No significant difference between conditions was observed.

There was a significant main effect of event (*F*[1,42]= 96.0, *p* < 0.001, η^2^=0.694), but no significant main effect of condition (*F*[1,42]= 0.05, *p* = 0.828, η^2^=0.001), and no significant condition by ROI interaction (*F*[1,42]= 0.86, *p* < 0.547, η^2^=0.020). We anyhow followed up the different areas to not miss effects in this exploratory study. This revealed enhanced functional processing in the individual areas during the olfactory compared to the trigeminal condition, revealing slightly higher activation in few areas, with highest effect size in the right amygdala (*F*[1,42]=1.56, *p*=0.218, η^2^=0.036), but none of the differences in any area reached significance threshold (see Figure 1).


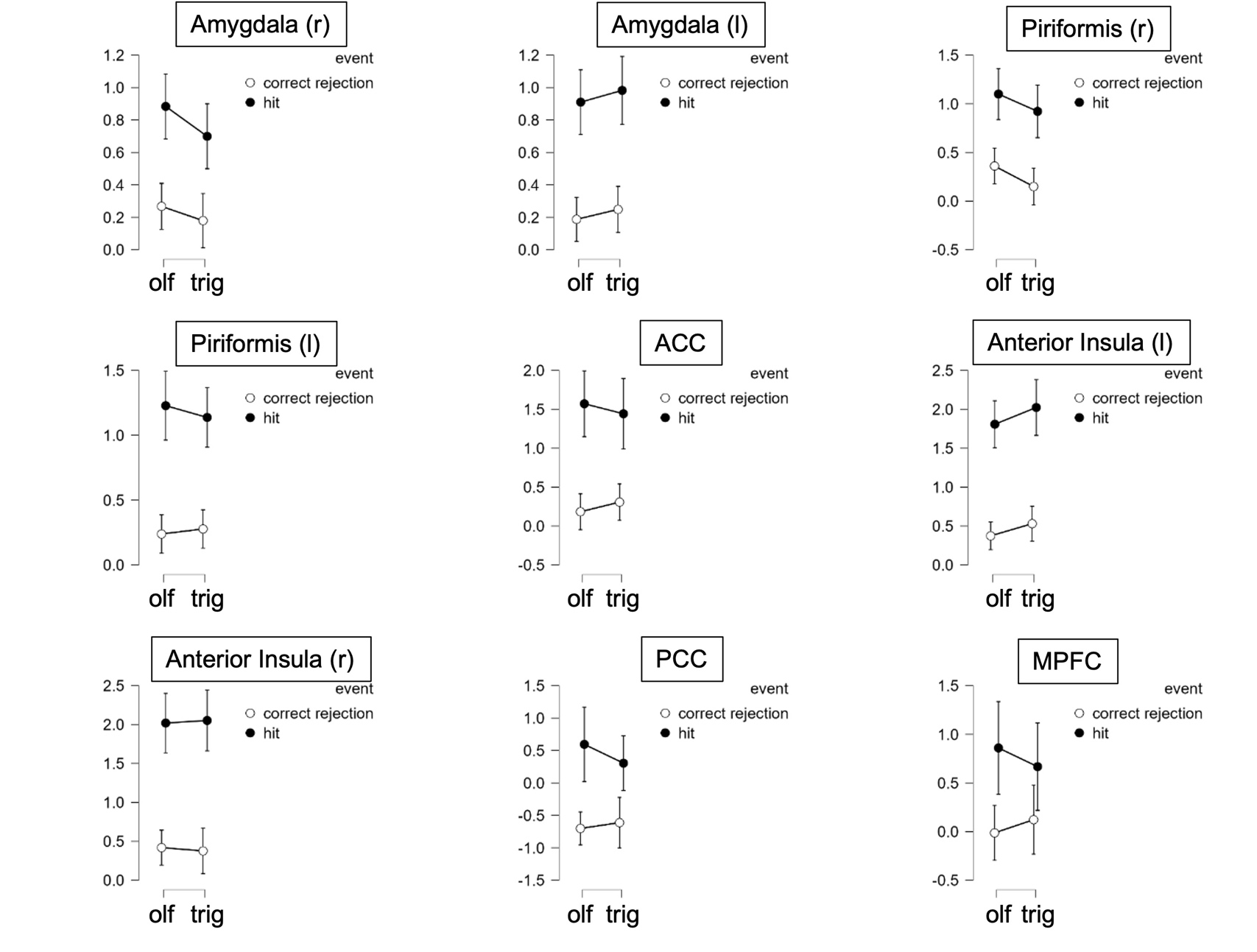


**Figure 1. ROI-Wise Comparison of Functional Processing Between Conditions.** Results of the oddball-paradigm are displayed for each region of interest (ROI), comparing functional processing between olfactory and trigeminal conditions. While exploratory analyses revealed slight differences in activation, including the largest effect size in the right amygdala (F[1,42] = 1.56, p = 0.218, η² = 0.036), no significant differences were detected between conditions for any ROI. ACC = anterior cingulate cortex. MPFC = medial prefrontal cortex. PCC = posterior cingulate cortex. r = right hemisphere. l = left hemisphere.

# *References*

1 Weiss T, Shushan S, Ravia A, Hahamy A, Secundo L, Weissbrod A *et al.* From Nose to Brain: Un-Sensed Electrical Currents Applied in the Nose Alter Activity in Deep Brain Structures. *Cereb Cortex* 2016; **26**: 4180–4191.

2 Hummel T, Sekinger B, Wolf SR, Pauli E, Kobal G. ‘Sniffin’ sticks’: olfactory performance assessed by the combined testing of odor identification, odor discrimination and olfactory threshold. *Chem Senses* 1997; **22**: 39–52.

3 Löwe B, Spitzer RL, Zipfel S, Herzog W. Auflage Manual 17.07. 2002; **9**: 44–45.

4 Poletti SC, Hausold J, Herrmann A, Witt M, Hummel T. Topographical distribution of trigeminal receptor expression in the nasal cavity. *Rhinology* 2019; **57**: 147–152.

5 Frasnelli J, Heilmann S, Hummel T. Responsiveness of human nasal mucosa to trigeminal stimuli depends on the site of stimulation. *Neurosci Lett* 2004; **362**: 65–69.

6 Meusel T, Negoias S, Scheibe M, Hummel T. Topographical differences in distribution and responsiveness of trigeminal sensitivity within the human nasal mucosa. *Pain* 2010; **151**: 516–521.

7 Scheibe M, Schulze S, Mueller CA, Schuster B, Hummel T. Intranasal trigeminal sensitivity: measurements before and after nasal surgery. *Eur Arch Otorhinolaryngol* 2014; **271**: 87–92.

8 Koeppel CJ, Herrmann T, Weidner K, Linn J, Croy I. Same salience, different consequences: Disturbed inter-network connectivity during a social oddball paradigm in major depressive disorder. *Neuroimage Clin* 2021; **31**: 102731.

9 Linden DEJ, Prvulovic D, Formisano E, Völlinger M, Zanella FE, Goebel R *et al.* The functional neuroanatomy of target detection: An fMRI study of visual and auditory/oddball tasks. *Cerebral Cortex* 1999; **9**: 815–823.

10 Jonathan D, Adrian P C, David J M, Karen D D. A multimodal cortical network for the detection of changes in the sensory environment. *Nat Neurosci* 2000; **3**: 277–283.

11 Stevens AA, Skudlarski P, Gatenby JC, Gore JC. Event-related fMRI of auditory and visual oddball tasks. *Magn Reson Imaging* 2000; **18**: 495–502.

12 Kim H. Involvement of the dorsal and ventral attention networks in oddball stimulus processing: A meta-analysis. *Hum Brain Mapp* 2014; **35**: 2265–2284.

13 Rozenkrants B, Polich J. Affective ERP processing in a visual oddball task: Arousal, valence, and gender. *Clinical Neurophysiology* 2008; **119**: 2260–2265.

14 Itz ML, Schweinberger SR, Schulz C, Kaufmann JM. Neural correlates of facilitations in face learning by selective caricaturing of facial shape or reflectance. *Neuroimage* 2014; **102**: 736–747.

15 Whitfield-Gabrieli S, Nieto-Castanon A. Conn: a functional connectivity toolbox for correlated and anticorrelated brain networks. *Brain Connect* 2012; **2**: 125–141.
